# Disinfection by Sulphur

**Published:** 1891-01

**Authors:** 


### DISINFECTION BY SULPHUR.

There has been much discussion recently respecting the efficiency of sulphur as a disinfectant for various infectious diseases, the efficiency of this method of disinfection having been denied by some physicians whose opinions have been widely quoted in the newspapers. Confirmatory of the results obtained by the State Board of Health of Michigan, we are glad to be able to quote the following from a work by Dujardin-Beaumetz, entitled "*Les Nouvels Médications* :"—

"Twenty grammes of sulphur to a cubic meter (1.53 lbs. per 1,000 cubic feet of air space), destroy the different micro-organisms in a moist state, but it is necessary to increase this dose if one wishes to destroy some organisms in a dry state. In fact since the last communications to the Academy, M. Bardet and myself, aided by M. Chambou, have continued these experiments upon micro-organisms in a dry state, and particularly upon vaccine virus. We have taken from the pustules of vaccinia, scabs which we have reduced to fine powder, and placed in chambers where were variable quantities of flowers of sulphur. When a dose did not exceed 20 grammes per cubic meter, the vaccine powder did not lose its properties, and one could, by inoculating animals and infants, obtain a vaccine eruption.

"With 30 grammes per cubic meter (2.297 pounds per 1,000 cubic feet of air space), the results obtained were uncertain, sometimes the powder losing its properties ; but when the dose is increased to 40 grammes per cubic meter (3.06 pounds per 1,000 cubic feet of air space), the inoculations are always inactive. So, then, for vaccine, and probably for variola, if one desires to destroy the contagious 'germs' in a dry state, it is necessary to double the dose of 20 grammes which we have already fixed.

"According to the experiments of Vallin and of Legouest, 20 grammes are sufficient for typhoid fever, while, according to Vallin, 40 grammes are necessary for the microbe of tuberculosis."

